# Soluble Fn14 Is Detected and Elevated in Mouse and Human Kidney Disease

**DOI:** 10.1371/journal.pone.0155368

**Published:** 2016-05-12

**Authors:** M. Nusrat Sharif, Gabriela Campanholle, Eva E. Nagiec, Ju Wang, Jameel Syed, Shawn P. O’Neil, Yutian Zhan, Karrie Brenneman, Bruce Homer, Hendrik Neubert, Riyez Karim, Nick Pullen, Steven M. Evans, Margaret Fleming, Priya Chockalingam, Lih-Ling Lin

**Affiliations:** 1 Inflammation and Immunology Research Unit, Pfizer Inc., Cambridge, MA, United States of America; 2 Pfizer Worldwide Drug Safety Research and Development, Cambridge, MA, United States America; 3 Pfizer Worldwide Drug Safety Research and Development, Andover, MA, United States of America; 4 Department of Pharmacokinetics, Dynamics and Metabolism, Pfizer Inc., Andover, MA, United States of America; 5 Global Biological Technology, Pfizer Inc., Cambridge, MA, United States of America; Instituto Nacional de Ciencias Medicas y Nutricion Salvador Zubiran, MEXICO

## Abstract

The cytokine TWEAK and its cognate receptor Fn14 are members of the TNF/TNFR superfamily and are upregulated in tissue injury to mediate local tissue responses including inflammation and tissue remodeling. We found that in various models of kidney disease, Fn14 expression (mRNA and protein) is upregulated in the kidney. These models include: lupus nephritis mouse models (Nephrotoxic serum Transfer Nephritis and MRL.Fas^*lpr/lpr*^), acute kidney injury models (Ischemia reperfusion injury and Folic acid injury), and a ZSF-1 diabetic nephropathy rat model. Fn14 expression levels correlate with disease severity as measured by disease histology. We have also shown for the first time the detection of soluble Fn14 (sFn14) in the urine and serum of mice. Importantly, we found the sFn14 levels are markedly increased in the diseased mice and are correlated with disease biomarkers including proteinuria and MCP-1. We have also detected sFn14 in human plasma and urine. Moreover, sFn14 levels, in urine are significantly increased in DN patients and correlated with proteinuria and MCP-1 levels. Thus our data not only confirm the up-regulation of Fn14/TWEAK pathway in kidney diseases, but also suggest a novel mechanism for its regulation by the generation of sFn14. The correlation of sFn14 levels and disease severity suggest that sFn14 may serve as a potential biomarker for both acute and chronic kidney diseases.

## Introduction

Tumor necrosis factor-like weak inducer of apoptosis (TWEAK, Apo3L, and TNFSF12) is a cytokine that belongs to the TNF superfamily. TWEAK binds and activates fibroblast growth factor-inducible-14 (Fn14, TWEAK receptor, TNFRSF12A, CD266), a TNF receptor superfamily (TNFRSF) protein [1–3]. In recent years, accumulating evidence has suggested a role for TWEAK activation of Fn14 receptor in the pathogenesis of acute and chronic kidney injury, contributing to both glomerular and tubulointerstitial damage in non-immune and immune-mediated kidney diseases [4–12].

The human TWEAK gene encodes a type II transmembrane glycoprotein of 249 amino acids. Proteolytic processing of membrane TWEAK generates soluble TWEAK (156-amino acids) [13–15]. TWEAK is broadly expressed, especially at relatively high levels in monocytes and macrophages. In the kidneys, both resident kidney cells and infiltrating leucocytes express TWEAK [9, 12, 16–19]. TWEAK expression levels in tissue and in circulation have been associated with various diseases including kidney (AKI) and autoimmune diseases (RA, SLE, IBD). Fn14 is the only characterized TWEAK receptor that can transduce both soluble and membrane TWEAK [13, 20]. Fn14 was originally described as an immediate-early response gene regulated by growth factors in fibroblasts [21, 22]. The human Fn14 gene encodes a type I transmembrane protein (129 amino acids) that is processed into a mature protein (102 amino acids). The extracellular domain (53 amino acids) contains a cysteine-rich domain necessary for TWEAK binding [23]. The intracellular domain (29 amino acids) of Fn14 lacks a death domain. However, it contains TNFR-associated factor (TRAF)-binding sites that can initiate the signaling events through recruitment of TRAF2/5 and activation of IKK and MAP kinase pathways [24, 25]. TWEAK can also bind the monocyte/macrophage scavenger receptor CD163. TWEAK binding to CD163 results in TWEAK internalization, but not in signaling. This suggests that CD163 behaves as a TWEAK scavenger receptor [26–28]. However, it should be noted that the role of CD163 in regulating TWEAK pathway remains controversial as Fick et al, have reported no evidence for an interaction of CD163 and TWEAK at biologically meaningful concentrations [29].

In healthy tissues Fn14 expression is low; however, cellular Fn14 levels are increased in response to stress or injury [12, 30–32]. In kidney, renal tubular epithelial cells, mesangial cells, podocytes and arterioles express Fn14 [9, 33–37]. In addition, leukocytes, such as macrophages, may express Fn14 and CD163 [27]. TWEAK regulates cellular events with potential pathophysiological relevance for kidney injury such as cell death, proliferation, differentiation, migration, inflammation, neoangiogenesis and tissue regeneration [38, 39]. Several studies have supported the pathogenic role of TWEAK/Fn14 pathway by using mouse knockout or antibody blockade in various mouse models of kidney diseases including AKI and chronic kidney disease (CKD). In particular TWEAK antibody and Fn14 KO studies have demonstrated a protective role in various SLE/LN models including NTN, MRL/lpr and cGvHD models [6, 10, 31, 40, 41]. In addition to kidney diseases, TWEAK/Fn14 has a potential role in the diseases of different organs, including heart, skeletal muscles, the central nervous system, liver and gut [42–51]. Indeed, several reports have provided data to demonstrate that tissue expression of TWEAK and Fn14 as well as circulating TWEAK levels are associated with these diseases in both mouse models and human patients [12, 31, 32, 52, 53]. Interestingly a decrease in sTWEAK may be due to overexpression of Fn14 and an increase in CD163-expresssing macrophages within injured tissue. Fn14 and TWEAK antibodies have been in the clinical development for treating cancer and lupus nephritis (such as BIIB023 TWEAK antibody for lupus nephritis) [31].

Many TNF receptors are shown to undergo proteolytic cleavage in the extracellular domain upon cellular activation. This shedding event generates soluble TNF receptor (sTNFR) that can be found in the circulation and in urine of patients with kidney diseases. In particular, the level of soluble TNFαR is associated with the progression of various diseases. For example, the levels of both TNFαR, sTNFR-1 and sTNF-R2, have been associated to progression of renal failure, end stage renal disease and mortality in early stages of CKD, mostly in the context of diabetic nephropathy [54]. In contrast to the well documented sTNF-R, the generation of soluble Fn14 (sFn14) has not been formally reported. The objective of this study was: 1) to confirm the upregulation of Fn14/TWEAK and 2) to investigate the presence of sFn14 and its association with kidney diseases. Fn14 is upregulated in tissue injury and we wanted to determine whether or not Fn14 is shed in disease and whether it can be a biomarker for kidney injury. Here we report that soluble Fn14 is generated and its levels are increased in various mouse models of kidney diseases. Importantly, we also observed that the sFn14 levels are increased in the urine from diabetic nephropathy patients. These data suggest that sFn14 may reflect kidney injury and inflammation in both acute and chronic kidney diseases; thus sFn14 level may be developed as a potential biomarker for the progression of kidney diseases. In addition, determination of sFn14 levels should be considered when evaluating the pharmacokinetic and pharmacodynamic effect of Fn14 or TWEAK antibody.

## Materials and Methods

### Animal Models

Studies were conducted in accordance with the National Institutes of Health Guide for the Care and Use of Laboratory Animals. All *in vivo* procedures were conducted under animal use protocols approved by Pfizer’s Institutional Animal Care and Use Committee (IACUC) in accordance to the Animal Welfare Act. Death was not an acceptable endpoint, thus moribund animals were removed from the studies and humanely euthanized according to a standard operating protocol (SOP) with defined objective clinical signs and appropriate timelines.

### Nephrotoxic Serum Nephritis Model (NTN Model

To induce Nephrotoxic serum nephritis, 129Sv/EvTac male mice (8–9 week of age; weighing 24-26g) were acquired from Taconic Farms and allowed to acclimate at least 7 days prior to the study. Mice were housed in a specific pathogen free (SPF) barrier facility on a 12:12h light: dark cycle with temperature, humidity and frequency of air changes in compliance with The Guide. Mice were provided standard rodent chow (Harlan 8640) and tap water *ad libitum* and housed under standard conditions. Chow was provided in standard, commercially available feeder devices. Mice were usually group housed in standard, transparent polycarbonate "shoe-box" style cages in alignment with The Guide. All cages were inspected on a daily basis and changed at least twice weekly to ensure animal access to clean, dry bedding as well as enrichment such as nestlets and igloos. At times, mice were singly housed in metabolic cages, where mice were provided Nalgene igloos, to enable urine collection.

Prior to study inception, mice were placed into weight-matched treatment groups. Control group had usually 6–8 mice, while the NTS treatment groups comprised 8–10 mice. Mice had body weights collected and health observations made daily following study inception.

Owing to the severity of nephrotoxic serum nephritis <10% of animals did not reach study endpoints and were humanely euthanized.

For induction of Nephrotoxic serum nephritis mice were first primed with sheep IgG in complete Freund’s adjuvant (day 0). On day 5, mice received an *i*.*v*. injection of Sheep anti-Rat GBM serum. Mice were anesthetized with 2% isoflurane for tail vein injections of Sheep Anti-Rat Glomerular Serum. No further anesthesia/analgesia was provided. Blood and urine were collected on day 0, day 7, 14 and 21 for serological measurements. Mice were deeply anesthetized with 5% isoflurane, followed by thoracotomy to ensure death. Tissue samples were collected and formalin-fixed or snap-frozen in liquid nitrogen for later evaluation. One kidney from each mouse was fixed in buffered formalin, embedded in paraffin, and used for histopathology and immunohistochemistry analysis. The other kidney was snap-frozen in liquid nitrogen for RNA studies.

For the NTN model, the overall disease severity score for glomerulonephritis was determined semi-quantitatively using light microscopic evaluation of hematoxylin and eosin-stained kidney sections by a board-certified veterinary pathologist. The score included changes in glomeruli (e.g., tuft enlargement, mesangial expansion, capsular adhesions, crescent formation, and/or periglomerular fibrosis), tubules (e.g., intraluminal protein, luminal dilation, epithelial degeneration/ regeneration, and/or rare intraluminal cell debris), and/or the interstitium (e.g., mixed cell inflammatory infiltrates and/or fibrosis). The scores were based upon the approximate percentage of the tissue affected: 0 = absent; 1 = ~1–20%; 2 = ~21–40%; 3 = ~41–60%; 4 = ~61–80%; 5 = ~81–100%. Similarly, semi-quantitative scoring of Fn14 immunopositivity was based upon the approximate tissue area that showed an increase in Fn14 immunopositivity compared with control tissue. The increases were observed in glomeruli, vessel walls, tubular epithelium and the content of tubules and Bowman’s space.

### MRL.Fas^*lpr/lpr*^ (MRL/lpr) spontaneous SLE model of glomerulonephritis

Kidneys were isolated from 21 week old female MRL/lpr mice (Jackson labs) as well as age matched MRL/MpJ mice (comparable control strain that does not develop SLE). There were 20 animals per group (N = 20) and 40 animals in the study. As described for the NTN model, one kidney from each mouse was processed for histopathology and immunohistochemistry while the other kidney was snap-frozen in liquid Nitrogen for RNA studies.

### Folic acid (FA) induced AKI model

CD1 male mice (8–9 week of age; Charles River Laboratories, Wilmington, MA) received a single intraperitoneal injection of folic acid (Sigma) 250 mg/kg in 0.3 mol/L sodium bicarbonate or vehicle, and mice were euthanized 24 hours later and blood was taken for serum analysis. The control group had (N = 5) mice, while the FA treated group had 9–10 mice, N = 15 per study. Urine was collected from animals placed in metabolic cages during a 24 hour period before mice were sacrificed. Kidneys harvested at necropsy were processed in a manner similar to NTN model as described above.

Tubular injury and Fn14 distribution scores: 5 = 75–100% of cortical and medullary tubules affected; 4 = 50–75% of cortical and medullary tubules affected; 3 = 25–50% of cortical and medullary tubules affected; 2 = 10–25% of cortical and medullary tubules affected; 1 = <10% of cortical and medullary tubules affected; 0 = no readily detectable cortical and medullary tubules affected.

### Human samples

Plasma and urine samples were commercially obtained from Bioreclamation IVT. Samples were collected with patient’s written consent and the study was reviewed and approved by Institutional Review Board (Schulman Associates). All aliquots were kept at -80C until analysis. Sodium heparin plasma from 32 healthy individuals (age 40±12 years) was used for sFn14 and sTWEAK measurements. Random morning spot urine was collected from a separated cohort of 26 diabetic nephropathy patients (age 60±9years) and 10 age-matched healthy subjects. See S1 Table for patient characteristics. The urine supernatant was used for analysis.

### ELISA

Serum and urine concentrations of mouse and rat soluble Fn14 (sFn14) were measured by species specific, sandwich enzyme-linked immunosorbent assays (ELISA; RayBiotech, CA). Difference in the molar mass of assay kit calibrator and natural soluble Fn14 was accounted for in calculations of serum and urinary concentrations of sFn14.

Urine concentrations of mouse MCP-1 and RANTES were measured by specific ELISAs, following manufacturer's recommendations (Quantikine, R&D Systems Inc, Minneapolis, MN). Assays were quantified at 450 nm in Spectramax M2 microplate reader (Molecular Devices, Sunnyvale, CA) and data were interpolated from the respective standard curves (dynamic range for Fn14 assay: 0.1–25 ng/ml; MCP-1assay: 7.8–1000 pg/ml; and for RANTES assay: 3.9–500 pg/ml) using SoftMax Pro software (version 5.2, Molecular Devices, Sunnyvale, CA). Urine samples required minimal dilution of 2-fold for MCP-1 and 4-fold for RANTES assessment, to reduce sample matrix interference. Urinary concentrations of analytes were expressed as ng/mg creatinine. Nonparametric statistics were used, Kruskal-Wallis’ one-way analysis of variance with Dunn’s multiple comparison test.

For quantitative determination of Fn14 in human plasma and urine, a newly developed sandwich ELISA was used. The assay consisted of a two non-competing anti-Fn14 monoclonal antibodies, PDL192 and 4A10 for capture and detection. PDL192 has been described previously [20] and was made in-house. 4A10 mouse mAb was generated by immunizing Balb/c mice with 300.19 cells overexpressing human FN14 followed by immunization with purified FN14 ectodomain protein. The specificity of 4A10 was demonstrated by specific binding to the FN14 ectodomain protein and increased binding to FN14-overexpressing cells as compared to the parental CHO cell lines based on flow cytometry (data not shown).

The detection antibody was labelled with biotin. Recombinant human Fn14 was used as standard. Color was developed with horseradish peroxidase (HRP) and 3, 3’, 5, 5’-Tetramethylbenzidine TMB (R&D system) and read at 450nm. The assay dynamic range was 0-2000pg/ml, with lower detection limit of 125pg/ml. Dilution of plasma and urine were linear and recovery ranged from 95 to 115% for spiked samples.

Human TWEAK (ebioscience BMS2006INST) and human MCP-1 (R&D #DCP00) in plasma and urine were measured with commercially available ELISA kits. Urinary levels of MCP-1, TWEAK and Fn14 were normalized by urine creatinine. Results were expressed in ng per mg of creatinine. Urine creatinine and protein were measured using the clinical chemistry system (Siemens ADVIA 1800).

### Quantitative Reverse Transcription (qRT)–PCR

RNA was isolated by RNeasy Mini Kit (Qiagen, Germantown, MD), and was reverse-transcribed qScript™ One-Step qRT-PCR Kits (Quanta Biosciences, Gaithersburg, MD). Real-time PCR was performed on an ABI Viia7 PCR system (Applied Biosystems, Foster City, CA) using the DeltaDelta Ct method. Expression levels are given as ratios to glyceraldehyde-3-phosphate dehydrogenase. Pre developed primer and probe assays for Fn14 and GAPDH were from Applied Biosystems.

### Immunohistochemistry (IHC) for Fn14

IHC for Fn14 was performed on 4 μm sections of formalin-fixed, paraffin-embedded (FFPE) mouse and human kidney tissue, using a Discovery XT automated immunostainer (Ventana Medical Systems, Inc., Tucson, AZ). Sections were baked and deparaffinized prior to heat-induced epitope retrieval in EDTA buffer (CC1, Ventana) and blocking for non-specific protein binding (S-Block, Ventana). Tissues were incubated with either anti-TWEAKR (Fn14) antibody (ab109365-2, abcam, Cambridge, MA) or negative control (Rabbit IgG, Vector Labs, Burlingame, CA), and primary antibody was detected with OmniMap anti-rabbit HRP multimer and ChromoMap DAB detection kits (Ventana). Sections were counterstained with hematoxylin and slides were cover slipped using a synthetic mounting medium.

### Measurement of Fn14 in human urine and human and mouse serum by immunoaffinity liquid chromatography tandem mass spectrometry (IA-LC-MS/MS)

Soluble FN14 was detected from human urine, normal human serum and C57/BL6 serum using an immunoaffinity liquid chromatography tandem mass spectrometry (IA-LC-MS/MS) assay. A 20 μL of serum or 200 μL aliquot of urine aliquot was combined with 1 μg biotinylated anti-FN14 monoclonal antibody (p4A8, described in reference 20 and produced in our lab) prior to dilution with PBS to 700 μL and incubation overnight at 4°C. 20 μL streptavidin coated magnetic beads (DynaBeads SA T1, Invitrogen Life Technologies, Carlsbad, CA) were incubated at room temperature for 1 hour. The beads were removed from the samples and washed twice with 0.05% CHAPS/PBS buffer followed by one wash with PBS. Bound sFN14 was eluted from the beads with two 70 μL aliquots of 25 mM HCl and collected in a fresh 1 mL Protein LoBind 96-deep well plate containing 30 μL of 2 M Tris–HCl, pH 8.0. Reduction was performed by the addition of 15 μL of 75 mM TCEP and incubation at 56°C for 45 min, followed by addition of 15 μL of 150 mM iodoacetamide and incubation in the dark at RT for 30 min to complete the alkylation. Samples were then digested overnight at 37°C using 1 μg of trypsin/Lys-C (Promega).

A Dionex Ultimate 3000 system was configured with a WPS-3000 autosampler, one micro-pump and one pump capable of nanoflow rates. For the digested immunoprecipitates from serum preparations, a PepMap300 C18 pre-column (5 × 0.3 mm, 5 μm, 300 Å, Thermo) and a PepMap C18 analytical column (15 cm × 75 μm, 3 μm, 100 Å, Easy-Spray, Thermo) were held at 60°C. The total chromatography duty cycle was 60 min at a flow rate of 400 nL/min. Buffer A was 2% acetonitrile in 0.1% formic acid and buffer B was 98% acetonitrile in 0.1% formic acid. Samples were loaded onto the trap and then eluted onto the analytical column, where gradient separation started from 3% buffer B at minute 3 and increased to 35% at minute 42, then to 45% B at minute 45, 90% B at minute 47 held for 3 minutes, before returning to starting conditions of 3% B. Mass spectra were acquired with a Q Exactive (Thermo Scientific) in a data-dependent manner using a top-8 method. Full MS spectra were acquired at a resolution of 70,000 with maximum integration time of 100 ms and a target value of 3 × 10^6^ ions. The m/z range was from 300 to 2000. Peptide fragmentation was achieved via higher-energy collisional dissociation (HCD) set at 27 V of normalized collisional energy. MS/MS of selected peptides was acquired at 17,500 resolution, with a target value of 5 × 10^5^ ions and a maximum integration time of 90 ms. The isolation widths was 2.0 m/z.

For the digested immunoprecipitates from urine preparations, the sample was first loaded on a PepMap300 C18 pre-column (5 × 0.3 mm, 5 μm, 300 Å, Dionex) followed by chromatographic separation on a PepMap C18 RSLC nanocolumn (15 cm × 75 μm, 2 μm, 100 Å, Dionex). The analytical gradient was at a flow rate of 600 nL/min using Solvent A (0.1% formic acid in 2% acetonitrile) and Solvent B (0.1% formic acid in 90% acetonitrile). From 0 to 4 min, the solvent composition was held at 5% B and then ramped up from 5% to 40% B during 4 to 13.5 mins of the cycle, reaching 90% B at 16mins and then brought back to initial conditions of 5%B for re-equilibration. The eluate was introduced into a nanospray III source of a 5500 Qtrap mass spectrometer (AB Sciex) using a nanospray stainless steel emitter (50 mm × 30 μm ID, Proxeon, West Palm Beach, FL). The MS was operated by multiple reaction monitoring (MRM) in positive ion mode for detection of the FN14 tryptic peptide using the following instrument parameters were employed: Ion spray voltage: around 3700 V; nebulizing gas: 5 psi; curtain gas: 10 psi and Interface heater temperature: 180°C. Q1 and Q3 were operated in unit and low resolution, respectively. The transitions monitored for the target peptide, GSSWSADLDK were: Q1 533.2 (doubly charged precursor ion) to Q3 921.4 (y8+), 834.4 (y7+), 648.3 (y6+) and 561.2 (y5+).

## Results

### Increased kidney Fn14 expression in NTN and MRL/lpr mouse model of Nephritis

**Fig 1:**
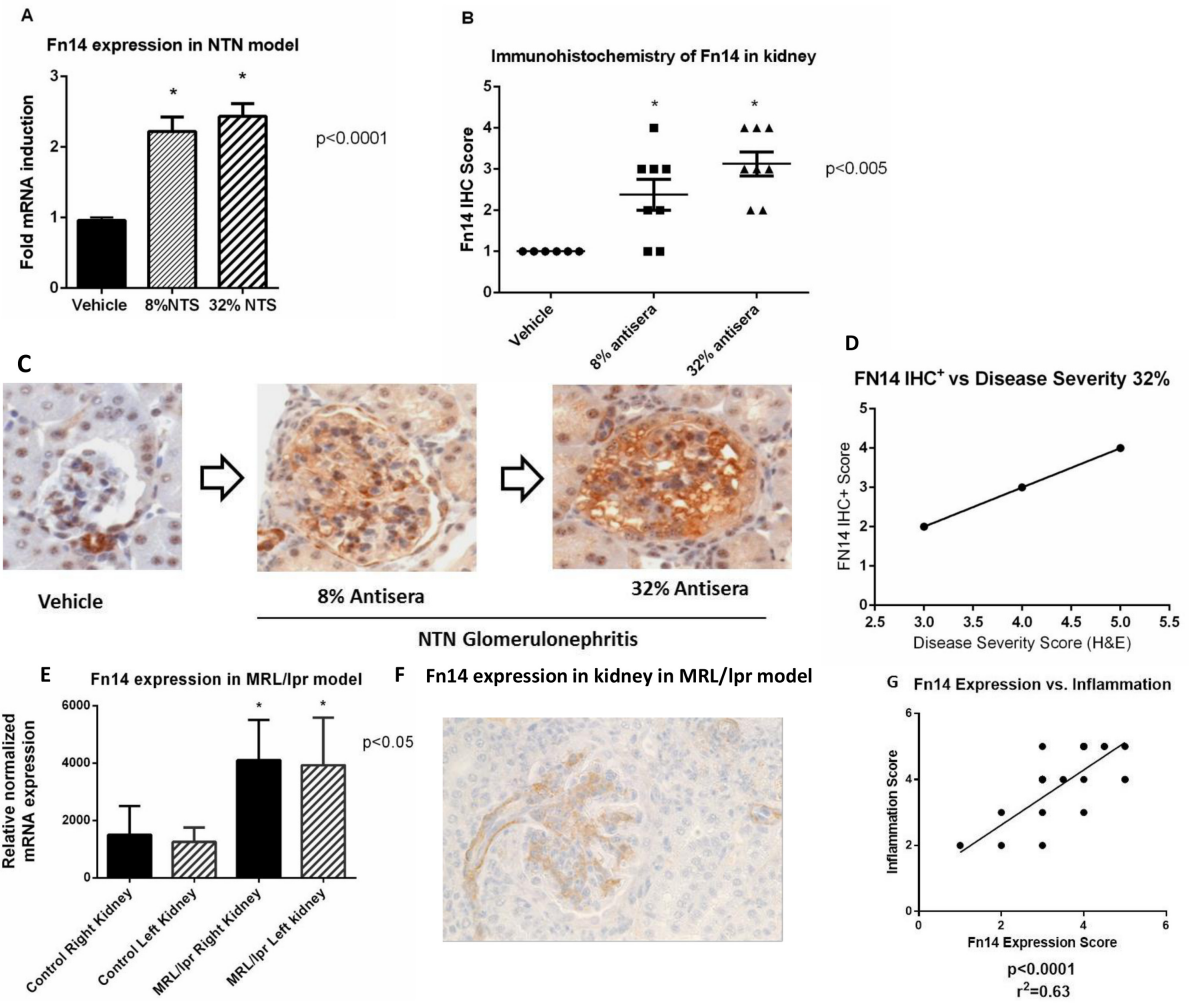
Fn14 expression in kidney. A) Fn14 mRNA expression is upregulated in disease vs vehicle in the NTN model (8% and 32% vs vehicle; p<0.001). B) Immunohistochemistry Score of Fn14 in kidney tissue in NTN model. The IHC staining of kidney shows increase in Fn14 protein consistent with the extent of injury in the NTN model (p<0.005). C) Fn14 IHC reveals progressive increase in Fn14 immunopositivity in glomeruli, vessels and tubular epithelium with disease progression in NTN model. D) Linear regression graph show a positive correlation between Fn14 expression by IHC- staining with the disease severity score by H&E in NTN model (*p = 0*.*0008*), R^2 =^ 0.914 E) Fn14 mRNA expression is increased with progression of disease in MRL/lpr mice spontaneous mouse model of Lupus Nephritis. Kidneys from MRL/lpr mice were harvested at 22 weeks of age. Aged matched controls were kidneys from MRL/MPJ mice- comparable strain that does not develop Lupus Nephritis. F) IHC showing Fn14 expression in kidney of MRL/lpr model. Fn14 expression was most prominent in arterioles and glomerulus. G) Strong association of Fn14 with inflammation score in MRL/lpr model. Linear regression plot showing correlation of Fn14 expression with inflammation (p<0.0001) and R^2 =^ 0.63.

To evaluate expression of Fn14 and its correlation with disease severity in nephritis, we used two different mouse models. The nephrotoxic nephritis (NTN) model was induced by immunizing mice with anti-rat glomerular serum diluted in PBS to final concentrations of 8% or 32% (serum concentration used was positively correlated with disease severity) (Fig 1).

Following induction of NTN Fn14 mRNA was upregulated when compared to control (8% and 32% vs vehicle (p<0.001) (Fig 1A). The increase in expression of Fn14 mRNA in the kidney tissue correlated with increased expression of Fn14 protein (p<0.005) detected by immunohistochemistry, on kidney sections (Fig 1B) and with disease progression, determined by presence and severity of microscopic lesions in glomeruli, vessels and tubular epithelium (Fig 1C). Fn14 protein expression was positively correlated (R^2^ = 0.914; p *= 0*.*0008*), with disease severity scores in NTN model (Fig 1D).

We also assessed the expression of the TWEAK/Fn14 pathway in pathogenesis of nephritis in the well-established MRL/lpr spontaneous mouse model of lupus. We found that kidney Fn14 protein and mRNA expression were both significantly increased in MRL/lpr mice at 22 weeks of age when compared to age matched control mice (MRL/MpJ) that do not develop SLE (Fig 1E–1F). Fn14 protein was increased in arterioles and glomerulus, however was not detected in the ducts or tubules by IHC (Fig 1F). The increase in both Fn14 mRNA and protein expression correlated well with increase in inflammation scores in the MRL/lpr model (Fig 1G) thus showing that Fn14 expression is associated with inflammation in the progressive nephritis of MRL/lpr mice.

Our results clearly demonstrate that Fn14 expression increases during both models of nephritis and correlates with disease severity.

### Detection of sFn14 in serum and urine from mouse in NTN model

**Fig 2:**
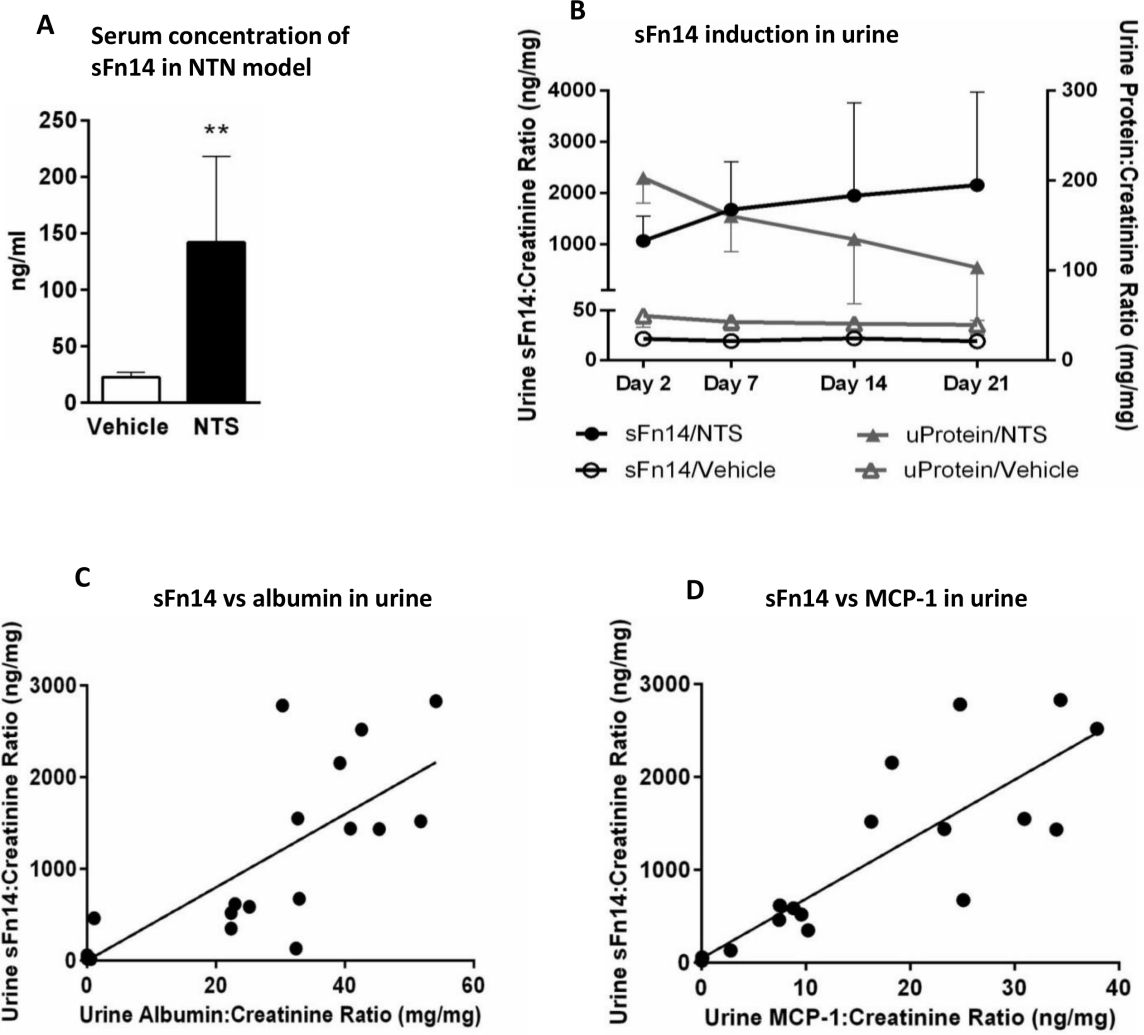
Fn14 concentration is increased in NTN model of kidney injury. (A) Increased concentration of Fn14 was detected in serum of mice injected with nephrotoxic serum (NTS) (vehicle vs NTS-**p = 0.006, Mann-Whitney test). Serum analysis was performed at the end of the study at 21 days. (B) Rapid increase in Fn14 concentration in urine (closed circles) apparent two days post NTS injection, was accompanied by development of proteinuria (closed triangles), and remained elevated through the duration of the study. (C) Urinary Fn14 strongly correlated with urinary albumin (r = 0.8803; p<0.0001, Spearman’s test) and urinary MCP-1 (r = 0.8978; p<0.0001, Spearman’s test), on day 7 of NTN induction.

We next wanted to determine the soluble Fn14 (sFn14) levels. We reason that generation of sFn14 can result from the receptor shedding in response to activation similar to that observed with other TNF receptor family members [55], thus providing additional evidence for the pathway activation. Thus, we assessed the levels of sFn14 in the serum (terminal collection) and urine from mice in the NTN model. Mean concentration of sFn14 in the serum of vehicle treated mice was at 22.6 ng/ml. We observed several fold increase in concentration of sFn14 in the serum from mice injected with nephrotoxic serum where mean value of 142 ng/ml was measured on day 21 (**p<0.006; Mann Whitney test) (Fig 2A). Rapid development of proteinuria in this model was accompanied by a mark increase in urinary sFn14 levels on day 2 and elevated throughout the duration of the study (Fig 2B, 25 ng sFn14 in vehicle vs ~1,000 ng sFn14/mg creatinine in NTN mice). Additionally, strong correlation was observed between the levels of sFn14 and other disease biomarkers, such as urine albumin (r = 0.8803 Spearman’s test) (Fig 2C) and urine MCP-1 (r = 0.8978, Spearman’s test) (Fig 2D).

### Increased kidney Fn14 expression in Ischemia Reperfusion model (IRI) and Folic Acid (FA) model of Acute Kidney Injury (AKI)

To determine whether renal ischemia induces Fn14 and TWEAK upregulation *in vivo*, we measured mRNA expression of both genes in the injured kidney using a murine renal IRI model. The kidneys were collected after 22 minutes of ischemia followed by 24, 48 or 72 hour of reperfusion. Fn14 mRNA expression was upregulated at all-time points when compared to vehicle, with the maximal increase observed 24 hours post ischemia (S1A Fig). In contrast, TWEAK mRNA expression did not change at any time-point. We examined the Fn14 protein expression in ischemic kidney by IHC (S1B Fig) and found that the protein expression of Fn14 was increased at 24 hours, consistent with the increase in Fn14 mRNA. The Fn14 protein expression at 48 hours was considerably lower and declined almost to the baseline at 72 hours, consistent with the injury resolution time-point in the IRI model. In addition, Fn14 expression was localized mostly to the cortico-medullary junction of ischemic kidney (S1C Fig). The increase in Fn14 expression in this model is consistent with a previous report [56].

**Fig 3:**
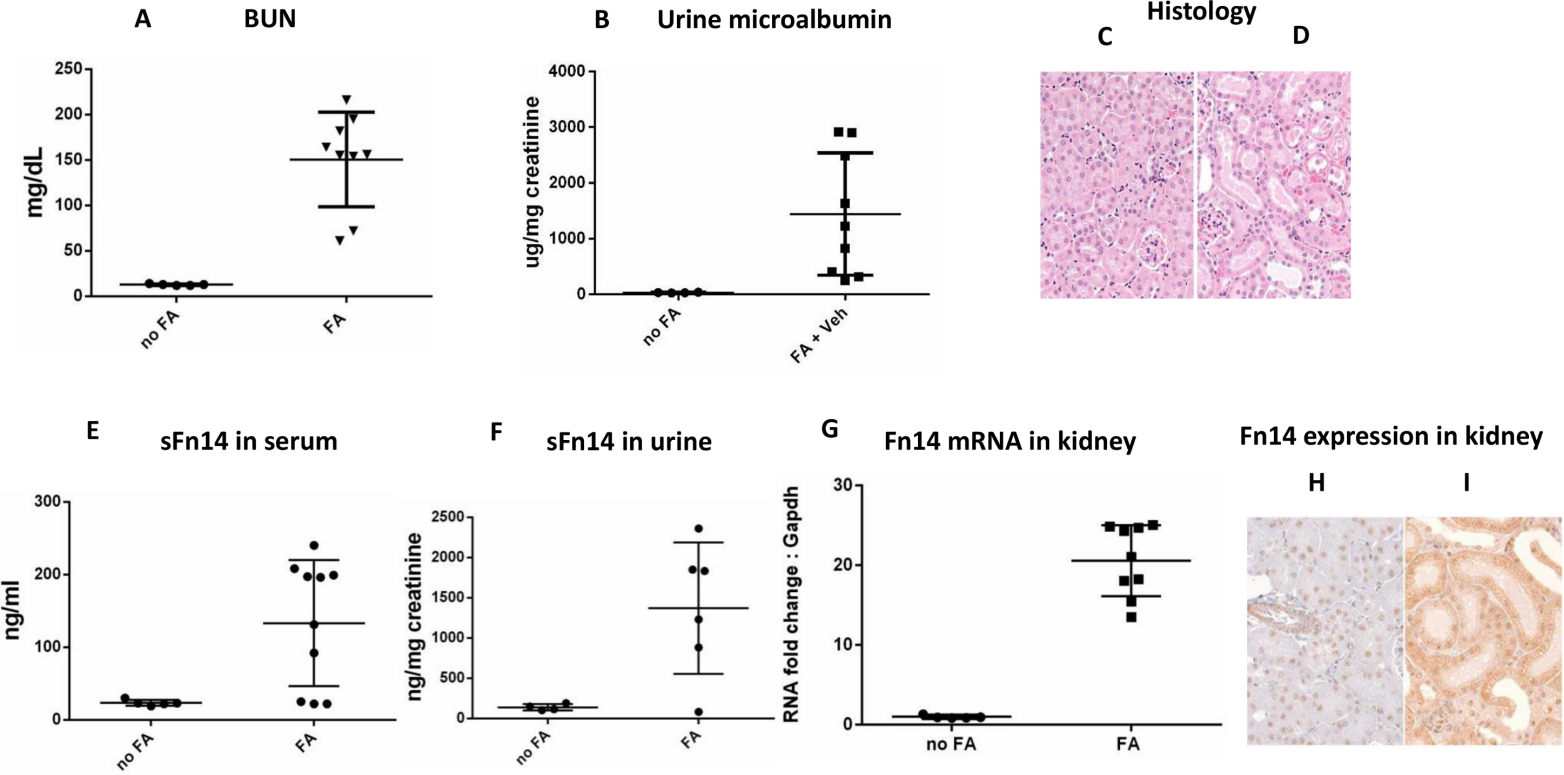
Fn14 is upregulated in Folic Acid induced acute kidney injury. Mice were systemically dosed with either vehicle or FA. (A) BUN and (B) Urine microalbumin are increased in FA treated mice 24h after dosing. Histology from (C) vehicle control kidney and (D) FA-treated mice show tubular dilatation and attenuation in FA dosed kidneys, indicating acute kidney injury. (E) sFn14 in serum and (F) sFn14 in urine is increased in FA-treated mice 24h after dosing. (G) qRT-PCR shows up-regulation of Fn14 mRNA in FA induced AKI kidneys. IHC from vehicle control kidney (H) and (I) FA-treated mice shows increased Fn14 immuno-reactivity in tubular epithelia of FA dosed mouse kidneys.

To determine whether Fn14 was upregulated in other models of AKI we used Folic Acid (FA) model. Acute tubular injury was induced by *i*.*p*. administration of 250mg/kg folic acid (Fig 3). Real time indicators of kidney injury, blood urine nitrogen (Fig 3A), urinary microalbumin (Fig 3B) and serum creatinine (data not shown) were increased 24 hours after folic acid dosing. The histological hallmarks of FA-induced AKI include tubular dilatation, attenuation and epithelial cell apoptosis in FA dosed kidneys (Fig 3C and 3D). The induction of AKI caused an increase in soluble Fn14 concentrations in both serum and urine (Fig 3E and 3F). Importantly, serum sFn14 levels are correlated with serum creatinine/BUN levels (S2 Fig), suggesting the association of sFn14 with kidney function. We have also observed a robust up-regulation of Fn14 transcripts in whole kidney lysate which correlates with sFn14 levels (Fig 3G and S2C Fig). Furthermore, in FA dosed kidney, the increase of Fn14 protein in kidney epithelium was detected by immunohistochemical analysis (Fig 3H and 3I).

### LC-MS/MS mass spectrometry studies of sFn14

In order to confirm the identity of sFn14 and specificity of the ELISA method for sFn14detection, we performed LC-MS/MS studies using sFn14 isolated from the serum of mice and human healthy volunteers. In the mouse sFn14 investigation, we detected a tryptic peptide corresponding to the ectodomain of mouse Fn14, thus confirming the identity of sFn14 (S3A Fig). Interestingly, for human sFn14, in addition to the ectodomain peptide, we detected a tryptic peptide originating from the cytosolic domain (S3B and S3C Fig). Further studies will be needed to confirm and fully characterize the sequence of the form of FN14 found in systemic circulation. The detection of the cytosolic sequences in human FN14 may suggest a novel mechanism for the generation of sFN14 in addition to receptor shedding.

### Fn14 and TWEAK in ZSF1 rat model of diabetic nephropathy (DN) and in human samples

**Fig 4:**
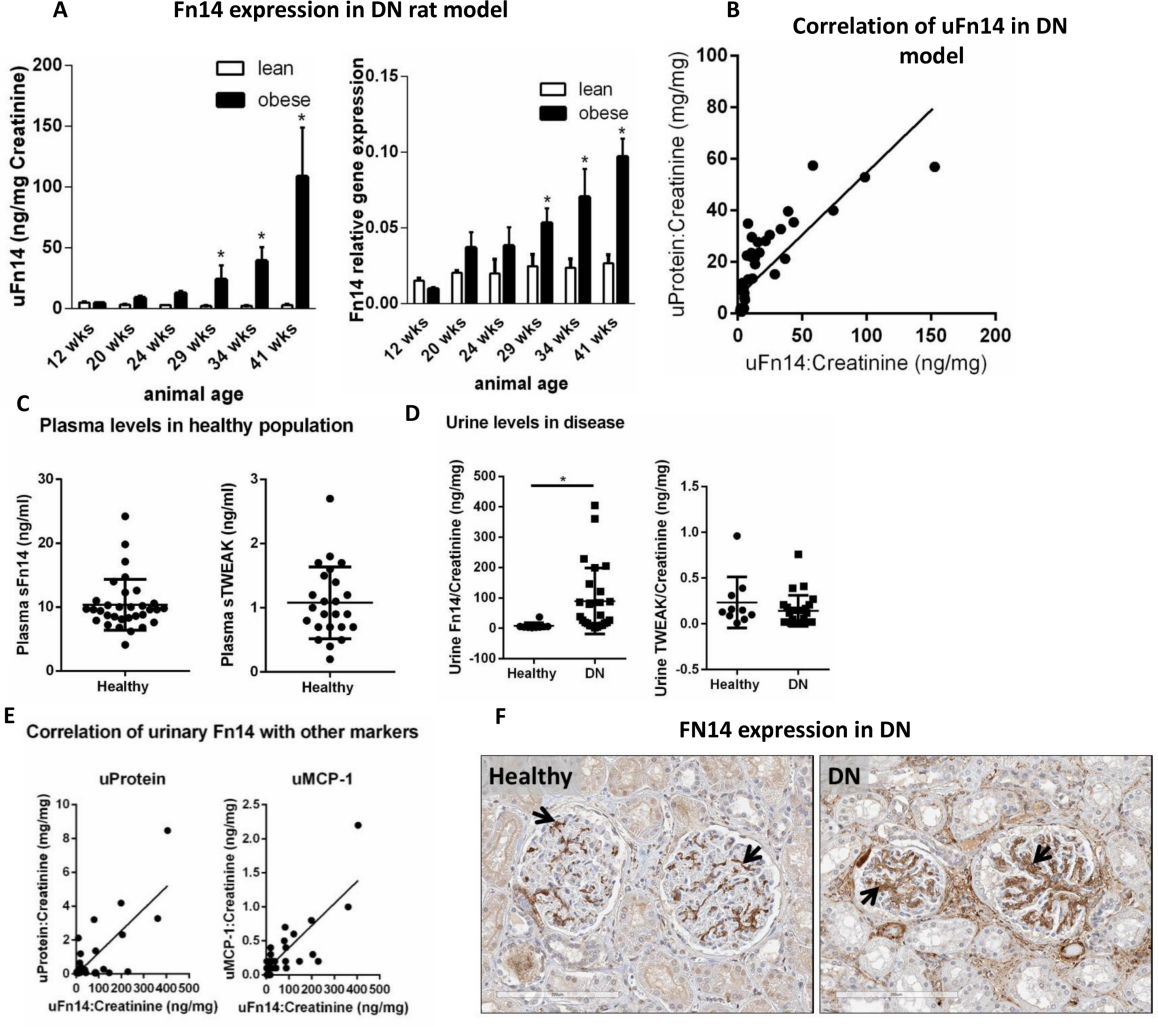
Fn14 and TWEAK expression in DN. A total of sixty male, 8-week-old ZSF1 obese and lean littermates were used in this study. (A) Urine levels of sFn14 and mRNA expression in kidney from ZSF1 lean (control) and obese (disease) rats (lean vs obese *p<0.0001). (B) Spearman correlation between urine sFn14 and total protein (r = 0.92, p<0.0001) in lean and obese ZSF1 rats (n = 5/group). (C) Plasma concentration of sFn14 (n = 32) and sTWEAK (n = 24) in healthy subjects. (D) Urine levels of sFn14 and sTWEAK in healthy controls (n = 10) and DN patients (n = 26). Data were normalized to urine creatinine, *p<0.0001, Mann-Whitney test. (E) Spearman correlation between urine sFn14, total protein (r = 0.5653, p<0.0005) and MCP-1 (r = 0.5790, p<0.0005), in healthy and DN patients (n = 36). (F) Immunohistochemistry showing Fn14 expression in kidney sections of healthy and DN patients. Fn14 is predominantly expressed in a fine reticular pattern that traces the glomerular mesangium and perivascular smooth muscle in the cortex of normal kidneys (arrows). In diabetic nephropathy, there is marked upregulation of Fn14 expression in the expanded mesangium of affected glomeruli (arrows).

Diabetic nephropathy is one of most prevalent kidney disease that includes both glomerular and tubular injury. In order to assess the expression of Fn14/sFn14 levels, we used a rat model of DN- ZSF1 rats. The ZSF1 rats turn obese and develop spontaneous Type 2 diabetes by 8 week of age, renal disease with proteinuria and renal failure by 20 weeks and die with end stage renal failure at 12 months of age [57]. Levels of sFn14 in urine as well as Fn14 mRNA were upregulated in obese rats when compared to lean controls during progression of disease (Fig 4A). Moreover, urine sFn14 positively correlated with levels of total protein (r = 0.92, p<0.0001, Fig 4B).

We extend our analysis of Fn14/TWEAK expression in human samples. We assessed the expression of FN14 and TWEAK in healthy subjects and in DN patients (see S1 Table for patient characteristics). Soluble FN14 levels were measured in plasma and urine by a validated ELISA developed in-house (see methods). The mean plasma level of sFN14 in 32 healthy individuals was 10±4 ng/ml, ten times higher than the TWEAK levels 1±0.5 ng/ml (Fig 4C). As described above, the identity of sFN14 was confirmed by detection of FN14 tryptic peptide sequences by mass spectrometry (S3 Fig). Furthermore, the relative high concentrations of sFN14 in human serum were also confirmed by a quantitative mass spectrometry method (unpublished data).

To understand the expression of this pathway in disease, sFN14 and TWEAK were measured in the urine of a small set of 10 healthy subjects and 26 DN patients by the ELISA method described above (Fig 4D). The levels of sFN14, normalized by urine creatinine, in DN patients were significantly higher than healthy controls (89±108 vs 8±10 ng/mg, respectively, p<0.0001). Urine sFN14 from DN patients can also be detected by the LC-MS/MS method (S3D Fig). The levels of sTWEAK were below detection range in 9 of the 36 urine samples analyzed, across healthy and disease subjects. For the purpose of statistical analysis and graph plotting, the lower detection limit value of the kit (0.015ng/ml) was attributed to those samples. No difference in urinary sTWEAK was observed between healthy controls and DN patients (0.2±0.2 vs 0.14±0.16 ng/mg, respectively).

We correlated urine levels of sFN14 with other urinary biomarkers (Fig 4E). Proteinuria in DN patients indicates increased glomerular permeability and renal damage. MCP-1 is a downstream chemokine regulated by Fn14/TWEAK pathway, and has previously been shown to be present in urine of DN patient [58]. The mean levels of urinary total protein and MCP-1 in control vs DN patients were (0.06±0.03 vs 1.1±1.9 mg/mg, p<0.05 and; MCP-1 0.12±0.09 vs 0.35±0.45, respectively). Importantly, urine sFN14 levels in healthy and DN patients positively correlates with levels of total protein (r = 0.5653, p<0.0005) and MCP-1 (r = 0.5790, p<0.0005) (Fig 4F). Urinary albumin was also up-regulated in DN patients (S1 Table) and correlated with sFn14 (r = 0.4580, p<0.005; data not shown).

We further evaluated whether FN14 and TWEAK are expressed in kidney tissue from DN patients. A representative immunohistochemistry image of kidney sections from control and DN patients (n = 2), shows that FN14 is expressed in glomerular mesangium in healthy kidneys and upregulated in the affected glomeruli (Fig 4F).

Together, this data suggests that Fn14/TWEAK pathway is regulated in DN patients. Even though a small set of human samples were used in this study, it suggests that FN14 might be a useful biomarker for kidney disease. Further evaluation is necessary and was out of the scope of this study. The high levels of circulating sFN14 in healthy individuals should be taken into account when considering it as a therapeutic target.

## Discussion

### Increased Fn14 expression in the diseased kidney

We have reported the increase of Fn14 mRNA and protein expression in the kidney of mouse models of lupus nephritis (NTN and MRL/lpr model) and acute kidney injury (folic acid and ischemia reperfusion), consistent with previous report [10, 12, 31, 32, 56]. Importantly, the Fn14 expression levels appear to correlate with disease severity in both NTN and MRL/lpr models (Fig 1D and 1G), suggesting that Fn14 pathway contributes to the disease. Indeed the causal role of Fn14 and its ligand, TWEAK, in these models have been supported based on the protective effect of either TWEAK antibody or Fn14 knockout mice in these models [10, 41]. Fn14 was upregulated in kidney of mice dosed with Folic acid (Fig 3). Our data is consistent with published reports that Fn14 expression increases during kidney injury and targeting TWEAK/Fn14 interactions might be beneficial in repair of acute and chronic kidney disease.

### Detection and elevation of sFn14 levels in rodent models of kidney diseases and in DN patients

We have reported for the first time the presence of soluble Fn14 in the urine and plasma of mouse models of kidney diseases and in the plasma and urine of healthy volunteer and patients with diabetic nephropathy. The identity of both human and mouse sFn14 is confirmed by the mass spectrometry study (S3 Fig). Importantly the sFn14 levels correlated with the disease severity in the NTN model (Fig 2) and in the folic acid AKI model (based on S2 Fig). Previous reports have shown that uTWEAK was upregulated in patients with active lupus nephritis and correlated with the activity of disease including a strong association with uMCP-1 levels [59]. We have found a similar association of urine sFn14 with disease features and uMCP-1 in NTN and AKI models, which, suggests that, sFn14 may offer a potential biomarker for kidney diseases.

There are several mechanisms that may contribute to the generation and elevation of sFn14 in the diseased tissues. Generation of sFn14 may come from induced expression of a soluble form of Fn14 gene or from alternative splicing of Fn14. However, the gene encoding corresponding sFn14 form has not been reported. It is also likely that generation of sFn14 may result from the shedding of Fn14 in response to a stimulus. Receptor shedding is common in the TNF receptor family. For example, TNFR1/2 is cleaved from the cell surface in response to various stimuli including its ligand, TNF [55, 60–67]. Soluble TNFR1/2 levels are also elevated in the kidney diseases and have emerged as robust predictors of the progression of diabetic kidney disease [68, 69]. Shedding and the resultant acute decrease in the number of receptor molecules on the cell surface may serve to transiently desensitize cells to the TWEAK action. In addition, the pool of soluble forms produced could function as decoy receptors, competing for the ligand with the cell surface receptors to attenuate the TWEAK activity. Moreover, it has been proposed that soluble receptors can stabilize and preserve circulating soluble TNF and thus function as TNF agonists [70].

The detection of the Fn14 sequences by MS studies provides additional evidence for the identity of sFn14 measured by the ELISA method. The detection of the sequences corresponding to the ectodomain is consistent with the receptor shedding mechanism, leading to the release of the extracellular domain of Fn14. The finding of the cytosolic sequences in human sFN14 suggests additional mechanisms. We speculate that the release of sFN14 can be mediated via receptor shedding, alternative splicing, cell death or secretion (such as via exosome and other microvesicles). These events can lead to the release of either ectodomain of sFN14 or a form that also includes the cytosolic sequences. Further studies to characterize the form of sFN14 and to elucidate the cellular events that trigger the release of sFN14 will be required to determine the mechanism of the generation and role of sFN14. Regardless the origin and role of sFn14, the significantly elevated sFn14 levels in instances of kidney disease provide a direct evidence for the activation of the TWEAK/Fn14 pathway in both acute and chronic kidney diseases.

### Regulation of TWEAK/Fn14 pathway in DN

We have reported that there is an association of urinary sFn14 with proteinuria and MCP-1, a potential disease biomarker, in the ZSF1 rat model of DN and in patients with diabetic nephropathy (Fig 4). This is the first report of elevation of sFn14 in preclinical DN model and in patients with DN. These data along with increased Fn14 expression (Fig 4A and 4F) suggest the upregulation of the TWEAK/Fn14 pathway in diabetic nephropathy. High levels of sFN14 in the urine from DN patients could be due to increased glomerular permeability or increased FN14 shedding in diseased kidney. The high levels of protein in the urine and increased FN14 protein expression in the kidney support both of these hypotheses. In this study, we did not find a significant change in urinary TWEAK levels in DN patients. Of note, decrease in serum TWEAK has been reported in patients with DN [71]. It is tempting to speculate that the decreased TWEAK levels are due to the elevation of sFN14, acting as a decoy receptor. Taken together, changes in TWEAK and sFN14 levels in DN support the dysregulation of this pathway in the disease.

## Conclusions

We have shown that the levels of Fn14 mRNA, protein and sFn14 are increased in various kidney diseases. In particular, the elevation of sFn14 levels appear associated with disease severity in the rodent models of kidney diseases and in DN patients. Additional studies are required to investigate the molecular mechanism and pathophysiological role of sFn14. Our results further support the hypothesis that TWEAK/Fn14 pathway is implicated in renal damage and sFn14 levels in serum and urine of patients with chronic kidney disease might be indicative of active ongoing disease. Taken together, our data suggest sFn14 as a potential biomarker for kidney diseases, and targeting the Fn14/TWEAK pathway may provide a potential therapeutic approach for treating renal diseases. Future work including longitudinal study in a large patient cohort is necessary to validate the utility of sFn14 as a potential disease biomarker. In addition, the high levels of circulating sFn14 and the complex mechanism of Fn14 regulation should be taken into account when considering it as a therapeutic target.

## Supporting Information

S1 Fig Fn14 Expression in IRI/AKI Model. (A) Fn14 mRNA increases 24 hours after IR, the levels of expression decrease at 48 and 72 hours but remain higher than the animals in the vehicle treated group. In contrast TWEAK expression remained about the same in this study. C57Bl/6J male mice 8–10 week old were used in IRI model. The control (vehicle) group had N = 8 mice, while each of the IR treatment groups had N = 15 mice. B) Fn14 protein expression by IHC- is increased at 24 hours, while expression slightly decreased at 48 hours, and subsequently declined at 72 hours, indicative of the time where injury resolves. C) IHC staining reveals Fn14 in the tubular injury at the cortico-medullary junction.
(PDF)

S2 Fig Corelation of sFn14 with serum creatinine/BUN and kidney Fn14 mRNA expression in FA/AKI Model. Linear regression graphs showing correlation of sFn14 with A) Creatinine, B) BUN and C) Fn14 mRNA in kidney in FA/AKI model. Serum sFn14 strongly correlated with serum Creatinine (R2 = 0.8285; p<0.0003 Spearman’s correlation) and BUN (R2 = 0.9; p<0.0001) (A and B). Urine Fn14 also correlated significantly (R2 = 0.7286; p<0.0021) with Fn14 mRNA expression in kidney (C).
(PDF)

S3 Fig Identification of sFn14 derived tryptic peptides by Immunoaffinity Liquid Chromatography Tandem Mass Spectrometry (IA-LC-MS/MS). Tandem mass spectra of sFN14 derived tryptic peptides acquired following FN14 immunoaffinity enrichment and trypsin digestion. (A) Mouse Fn14 tryptic peptide: 28EQAPGTSPC*SSGSSWSADLDK (*C carbamidomethylated, Uniprot ID Q9CR75) corresponding to the ectodomain of mouse Fn14. (B) Human FN14 tryptic peptide: 39GSSWSADLDK (Uniprot ID Q9NP84), corresponding to the ectodomain of FN14; (C) Human FN14 tryptic peptide: 108EKFTTPIEETGGEGC*PAVALIQ* (*C carbamidomethylated, *Q deamidated, Uniprot ID Q9NP84). Note this tryptic peptide originates from the cytoplasmic domain of FN14. (D) LCMS detection of Fn14 tryptic peptide (GSSWSADLDK) in the urine of human DN samples- Briefly 200ul of urine sample was incubated overnight with P4A8 antibody at 40C and tryptic peptide measured by LCMS assay.
(PDF)

S1 Table Clinical Characteristics of DN patients studied. (PDF)
