# Supplementary material for: Soluble Fn14 Is Detected and Elevated in Mouse and Human Kidney Disease
Source: PLoS One. 2016 May 12;11(5):e0155368. doi: 10.1371/journal.pone.0155368 (PMC4865213; doi:10.1371/journal.pone.0155368)
Supplement: S1 Fig — (A) Fn14 mRNA increases 24 hours after IR, the levels of expression decrease at 48 and 72 hours but remain higher than the animals in the vehicle treated group. In contrast TWEAK expression remained about the same in this study. C57Bl/6J male mice 8–10 week old were used in IRI model. The control (vehicle) group had N = 8 mice, while each of the IR treatment groups had N = 15 mice. B) Fn14 protein expression by IHC- is increased at 24 hours, while expression slightly decreased at 48 hours, and subsequently declined at 72 hours, indicative of the time where injury resolves. C) IHC staining reveals Fn14 in the tubular injury at the cortico-medullary junction. (PDF) [file pone.0155368.s001.pdf]

Figure S1: Fn14 Expression in IRI/AKI model

A Induction of Fn14 in mouse AKI/IRI kidney

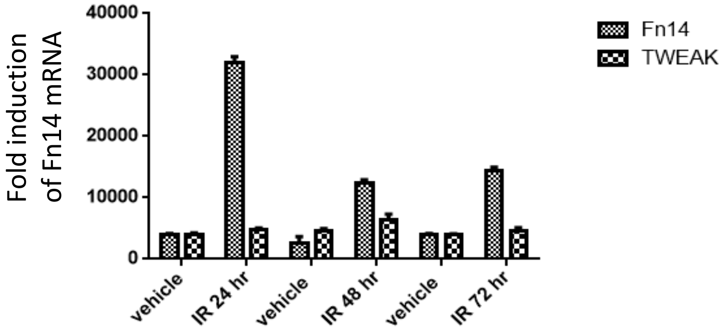

B Fn14 protein expression by IHC in AKI/IRI

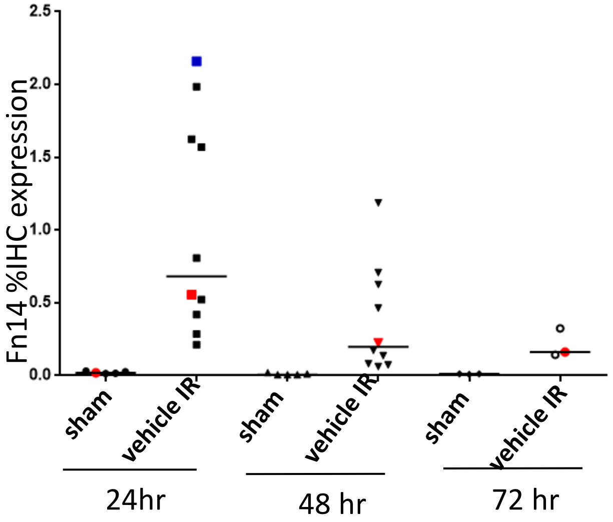

C IHC staining of Fn14 in IRI kidney

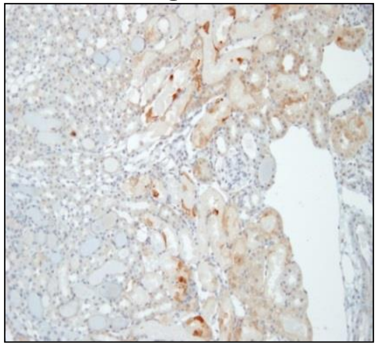

Figure S1: (A) Fn14 mRNA increases 24 hours after IR, the levels of expression decrease at 48 and 72 hours but remain higher than the animals in the vehicle treated group. In contrast TWEAK expression remained about the same in this study. C57Bl/6J male mice 8-10 week old were used in IRI model. The control (vehicle) group had N=8 mice, while each of the IR treatment groups had N=15 mice. B) Fn14 protein expression by IHC- is increased at 24 hours, while expression slightly decreased at 48 hours, and subsequently declined at 72 hours, indicative of the time where injury resolves. C) IHC staining reveals Fn14 in the tubular injury at the cortico-medullary junction.
