# Supplementary material for: Soluble Fn14 Is Detected and Elevated in Mouse and Human Kidney Disease
Source: PLoS One. 2016 May 12;11(5):e0155368. doi: 10.1371/journal.pone.0155368 (PMC4865213; doi:10.1371/journal.pone.0155368)
Supplement: S2 Fig — Linear regression graphs showing correlation of sFn14 with A) Creatinine, B) BUN and C) Fn14 mRNA in kidney in FA/AKI model. Serum sFn14 strongly correlated with serum Creatinine (R2 = 0.8285; p<0.0003 Spearman’s correlation) and BUN (R2 = 0.9; p<0.0001) (A and B). Urine Fn14 also correlated significantly (R2 = 0.7286; p<0.0021) with Fn14 mRNA expression in kidney (C). (PDF) [file pone.0155368.s002.pdf]

Figure S2: Correlation of sFn14 with Serum Creatinine/BUN and kidney Fn14 mRNA expression in FA/AKI model

A) Serum sFn14 vs Serum Creatinine

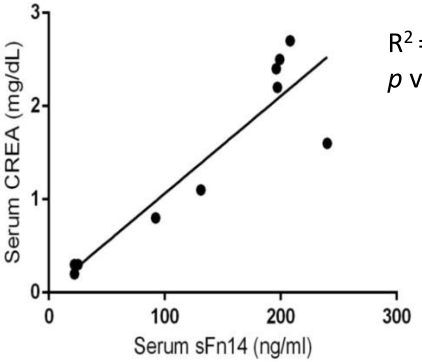

R^2 =0.8285
p value= 0.0003

B) Serum sFn14 vs Serum BUN

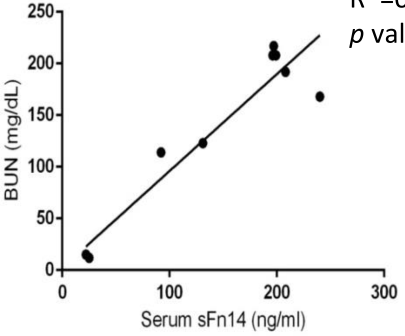

R^2 =0.90
p value <0.0001

C) Urine Fn14 vs kidney Fn14 expression

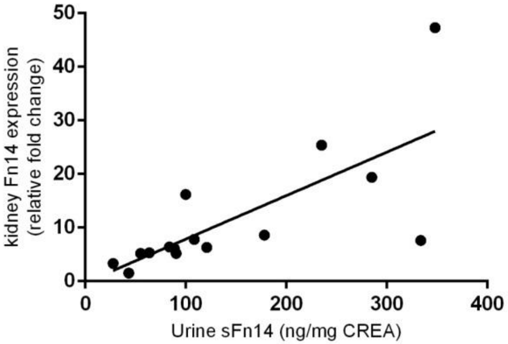

R^2 =0.7286
P value<0.0021.

Figure S2: Linear regression graphs showing correlation of sFn14 with A) Creatinine, B) BUN and C) Fn14 mRNA in kidney in FA/AKI model. Serum sFn14 strongly correlated with serum Creatinine (R2=0.8285; *p<0.0003* Spearman's correlation) and BUN (R^2=0.9; *p<0.0001*) (A and B). Urine Fn14 also correlated significantly (R^2 =0.7286; *p<0.0021)* with Fn14 mRNA expression in kidney (C).
