# Supplementary material for: Soluble Fn14 Is Detected and Elevated in Mouse and Human Kidney Disease
Source: PLoS One. 2016 May 12;11(5):e0155368. doi: 10.1371/journal.pone.0155368 (PMC4865213; doi:10.1371/journal.pone.0155368)
Supplement: S3 Fig — Tandem mass spectra of sFN14 derived tryptic peptides acquired following FN14 immunoaffinity enrichment and trypsin digestion. (A) Mouse Fn14 tryptic peptide: 28EQAPGTSPC*SSGSSWSADLDK (*C carbamidomethylated, Uniprot ID Q9CR75) corresponding to the ectodomain of mouse Fn14. (B) Human FN14 tryptic peptide: 39GSSWSADLDK (Uniprot ID Q9NP84), corresponding to the ectodomain of FN14; (C) Human FN14 tryptic peptide: 108EKFTTPIEETGGEGC*PAVALIQ* (*C carbamidomethylated, *Q deamidated, Uniprot ID Q9NP84). Note this tryptic peptide originates from the cytoplasmic domain of FN14. (D) LCMS detection of Fn14 tryptic peptide (GSSWSADLDK) in the urine of human DN samples- Briefly 200ul of urine sample was incubated overnight with P4A8 antibody at 40C and tryptic peptide measured by LCMS assay. (PDF) [file pone.0155368.s003.pdf]

Figure S3: Identification of sFN14 derived tryptic peptides by immunoaffinity liquid chromatography tandem mass spectrometry (IA-LC-MS/MS).

A Tryptic peptide of sFn14 in mouse serum

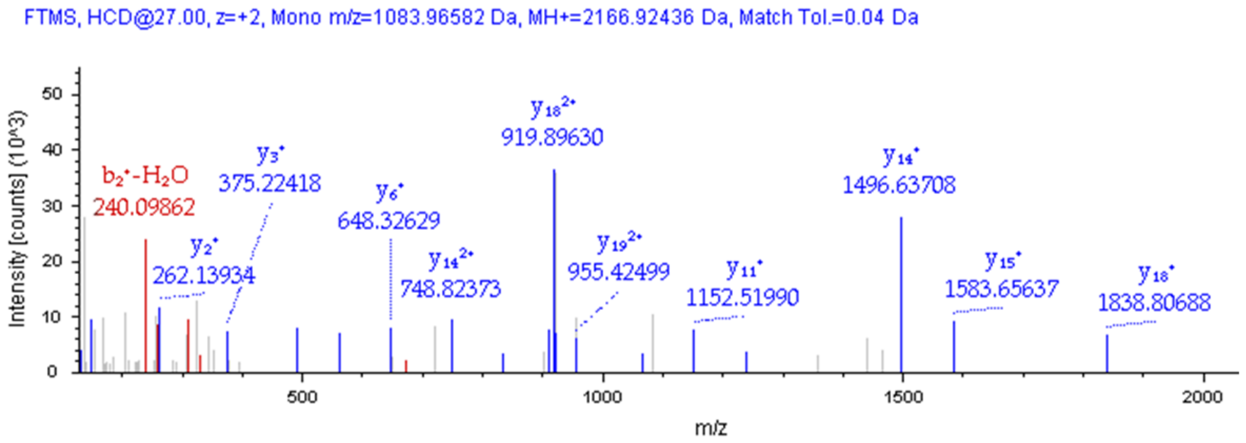

B Tryptic peptide of sFN14 in human serum

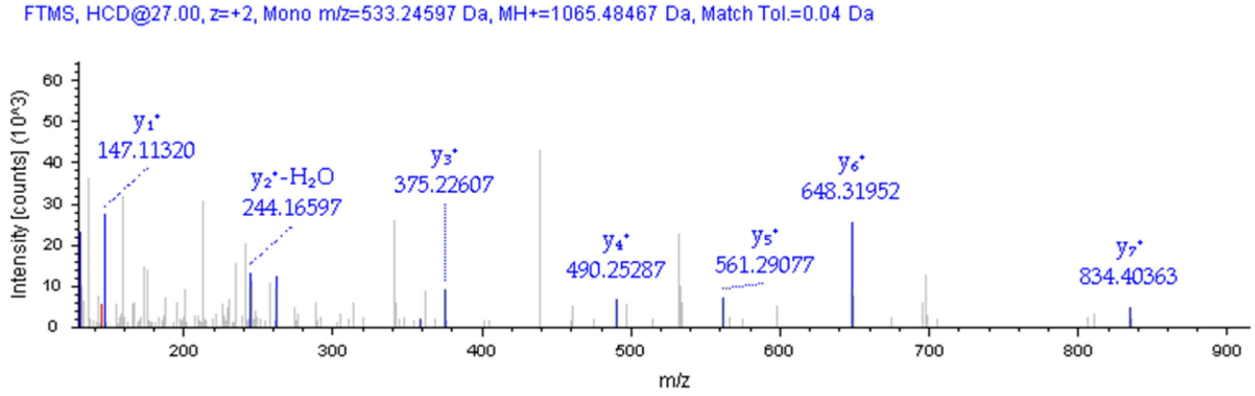

C Tryptic peptide of sFN14 in human serum

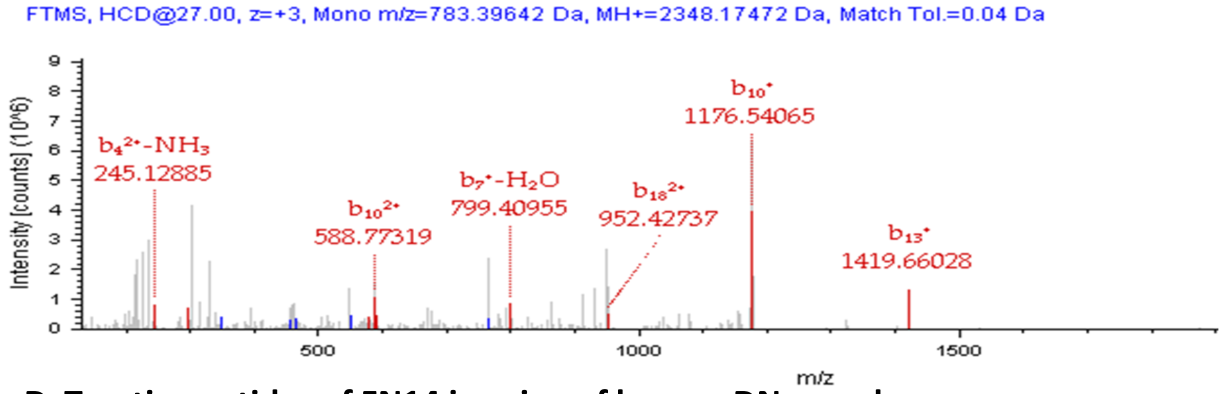

D Tryptic peptides of FN14 in urine of human DN samples

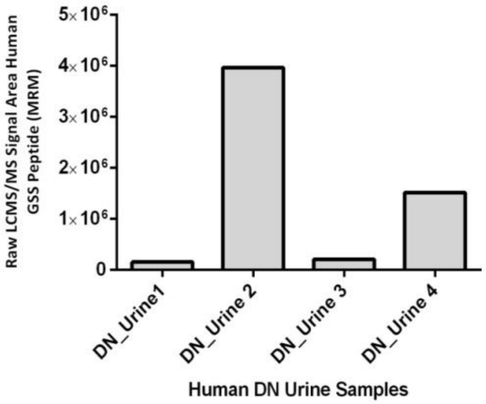

Figure S3: Tandem mass spectra of sFN14 derived tryptic peptides acquired following FN14 immunoaffinity enrichment and trypsin digestion. **(A)** Mouse Fn14 tryptic peptide: ^{28}EQAPGTSPC*SSGSSWSADLDK (*C carbamidomethylated, Uniprot ID Q9CR75) corresponding to the ectodomain of mouse Fn14. **(B)** Human FN14 tryptic peptide: ^{39}GSSWSADLDK (Uniprot ID Q9NP84), corresponding to the ectodomain of FN14; **(C)** Human FN14 tryptic peptide: ^{108}EKFTTPIEETGGEGC*PAVALIQ* (*C carbamidomethylated, *Q deamidated, Uniprot ID Q9NP84). Note this tryptic peptide originates from the cytoplasmic domain of FN14. **(D)** LCMS detection of Fn14 tryptic peptide (GSSWSADLDK) in the urine of human DN samples- Briefly 200ul of urine sample was incubated overnight with P4A8 antibody at 4^0C and tryptic peptide measured by LCMS assay.
