# Supplementary material for: Soluble Fn14 Is Detected and Elevated in Mouse and Human Kidney Disease
Source: PLoS One. 2016 May 12;11(5):e0155368. doi: 10.1371/journal.pone.0155368 (PMC4865213; doi:10.1371/journal.pone.0155368)
Supplement: S1 Table — (PDF) [file pone.0155368.s004.pdf]

Table S1: Clinical Characteristics of DN Patients Studied

Table S1. Patient characteristics						
**Sample matrix**	**Diagnosis**	**n**	**Age**	**Sex (male/female)**	**uAlbumin/creatinine ratio (ug/mg)**	**uProtein/creatinine ratio (mg/mg)**
Urine	Healthy	10	51.3±4.9	0/10	17.8±20.91	0.06±0.03
Urine	Diabetic nephropathy	26	59.8± 8.7	15/11	807.2±1386*	1.11±1.91*
Plasma	Healthy	32	39.9±12.7	27/5	N/A	N/A

* p<0.005, urine healthy vs DN
